# Supplementary material for: Factors influencing preoperative chest radiography request for elective endoscopic procedures among medical personnel
Source: PLoS One. 2020 Nov 13;15(11):e0242140. doi: 10.1371/journal.pone.0242140 (PMC7665807; doi:10.1371/journal.pone.0242140)
Supplement: S1 Appendix — (PDF) [file pone.0242140.s001.pdf]

Appendix

Questionnaire for assessment of knowledge and attitudes of medical personnel about preprocedural or preoperative requests for chest radiography

This questionnaire consists of 3 parts. Please answer all of the following questions according to your actual opinion. Your answers are valuable for our research on the knowledge, attitudes, and actual practices of medical personnel on preprocedural or preoperative requests for chest radiography. This research may assist with the development of appropriate guidelines for preprocedural or preoperative chest radiography requests at Siriraj Hospital. Please feel assured that your answers will be kept confidential, and they will not be individually analyzed.

Our research team recognizes that that this questionnaire is relatively long and therefore may take some time to complete. However, we greatly hope to get your cooperation.

Thank you very much for giving your time and making the effort to contribute to this research for the benefit of society.

Part1. General information

1. Age: ……………. years
2. Gender

   ❒ 1. Female ❒ 2. Male
3. Position

   ❒ 1. Resident ❒ 2. Fellow
4. Department

   ❒ 1. Anesthesiology ❒ 2. Surgery ❒ 3. Gastrointestinal Medicine
5. Year of training

   ❒ 1. 1st year ❒ 2. 2nd year ❒ 3. 3rd year

   ❒ 4. 4th year ❒ 5. 5th year
6. Have you ever attended any courses on the preoperative chest radiography?

   ❒ 0. No ❒ 1. Yes
7. Are you the person who decides to make requests for preoperative chest radiography?

   ❒ 0. No ❒ 1. Yes
8. How many patients have you examined and proceeded to endoscopic procedures during the past 3 months?

   ❒ 1. 0-10 ❒ 2. 10-20 ❒ 3. 20-30

   ❒ 4. 30-40 ❒ 5. > 40

Part 2. Basic knowledge about preprocedural or preoperative chest radiography

The following questions are designed to assess your knowledge. Your answers will not affect your current work. Please select your answer and mark ✓ in the ❒.

9. All patients who are undergoing surgery should have preprocedural chest radiography.

   ❒ 0. No ❒ 1. Yes

10. All patients aged over 45 years should have preprocedural chest radiography.

    ❒ 0. No ❒ 1. Yes

11. All patients scheduled for major surgeries (for example, kidney transplantation) should have preprocedural chest radiography.

    ❒ 0. No ❒ 1. Yes

12. All pediatric patients should have preprocedural chest radiography.

    ❒ 0. No ❒ 1. Yes

13. A 39-year-old male patient with atrial fibrillation is scheduled for hernioplasty. He should have chest radiography before the operation.

    ❒ 0. No ❒ 1. Yes

14. A 35-year-old woman with asthma is scheduled for colonoscopy. She has not developed active asthma for 1 year and therefore does not require any bronchodilators. Chest radiography should be required before the procedure.

    ❒ 0. No ❒ 1. Yes

15. A 20-year-old man has rheumatic heart disease. He does not have dyspnea and can walk up 2 flights of stairs. The patient is scheduled for mitral valve replacement. Chest radiography should be required before the operation.

    ❒ 0. No ❒ 1. Yes

16. A 50-year-old man has chronic obstructive pulmonary disease (COPD). He stopped smoking 10 years ago, and he has not had any COPD exacerbation that required hospitalization during the last 2 years. He is scheduled for transurethral resection of the prostate (TURP). Chest radiography should be required before the operation.

    ❒ 0. No ❒ 1. Yes

17. A 50-year-old female patient with chronic kidney disease (CKD) requiring hemodialysis 2 times/week is scheduled for cataract surgery. She does not have a history of pulmonary edema. Chest radiography should be required before the operation.

    ❒ 0. No ❒ 1. Yes

18. A 30-year-old woman with no underlying diseases is scheduled for thyroidectomy. Chest radiography should be required before the operation.

❒ 0. No ❒ 1. Yes

19. A 70-year-old man with ischemic heart disease is scheduled for pylorus-preserving pancreaticoduodenectomy. He does not have chest pain. However, he has to take a rest after walking up 1 flight of stairs. Chest radiography should be required before the operation.

❒ 0. No ❒ 1. Yes

20. A 20-year-old woman with no underlying diseases is complaining about off and on pain at the epigastrium. She is scheduled for esophagogastroduodenoscopy (EGD). Chest radiography should be required before the procedure.

❒ 0. No ❒ 1. Yes

21. A 35-year-old man with esophageal cancer is scheduled for esophagectomy with a gastric pull-up. Chest radiography should be required before the operation.

❒ 0. No ❒ 1. Yes

22. A 30-year-old woman with acoustic neuroma is scheduled for craniotomy with tumor removal. Chest radiography should be required before the operation.

❒ 0. No ❒ 1. Yes

23. A 6-hour term male neonate, post conceptual age (PCA) 37 weeks, is diagnosed with gastroschisis. He is scheduled for primary closure. Chest radiography should be required before the operation.

❒ 0. No ❒ 1. Yes

24. The Central region of Thailand is where tuberculosis is most prevalent.

❒ 0. No ❒ 1. Yes

25. During 2017, the incidence of tuberculosis in Thailand was 172 per 100,000 people.

❒ 0. No ❒ 1. Yes

26. Chest radiography is highly specific for tuberculosis.

❒ 0. No ❒ 1. Yes

Answer key for the knowledge questions

9: No. 10: No. 11: No. 12: No. 13: No. 14: No. 15: Yes. 16: Yes. 17: No. 18: Yes. 19: Yes.

20: No. 21: Yes. 22: Yes. 23: No. 24: No. 25: Yes. 26: No.

Part 3. Attitudes about preprocedural or preoperative requests for chest radiography

The following questions allow assessment of your opinions on preprocedural or preoperative requests for chest radiography. Please select your answer and mark ✓ in the ❒.

The answers are graded according to the level of importance, namely, extremely important (5), very important (4), moderately important (3), slightly important (2), and very slightly important (1). Please answer all of the questions.

27. How important is it to chest radiography requests to consider the following factors before an operation or endoscopic procedure?

27.1) History of pulmonary tuberculosis

❒ 5 ❒ 4 ❒ 3 ❒ 2 ❒ 1

27.2) History of heart disease

❒ 5 ❒ 4 ❒ 3 ❒ 2 ❒ 1

27.3) History of chronic obstructive pulmonary disease (COPD)

❒ 5 ❒ 4 ❒ 3 ❒ 2 ❒ 1

27.4) History of upper respiratory tract infection

❒ 5 ❒ 4 ❒ 3 ❒ 2 ❒ 1

27.5) Smoking

❒ 5 ❒ 4 ❒ 3 ❒ 2 ❒ 1

27.6) Healthy patient older than 45 years with no underlying diseases

❒ 5 ❒ 4 ❒ 3 ❒ 2 ❒ 1

28. A 40-year-old male patient with hypertension is diagnosed with acute appendicitis and will be scheduled for appendectomy. He can walk up 2 flights of stairs. He does not have a previous history of chronic cough, chest pain or weakness of extremities. How do the following factors affect your decision to request preoperative chest radiography?

28.1) Avoidance of operation cancellation by surgeons and anesthesiologists

❒ 5 ❒ 4 ❒ 3 ❒ 2 ❒ 1

28.2) Tuberculosis surveillance

❒ 5 ❒ 4 ❒ 3 ❒ 2 ❒ 1

28.3) Prevention of risks or complications during the operation

❒ 5 ❒ 4 ❒ 3 ❒ 2 ❒ 1

28.4) Prevention of prosecution should there be adverse events during the operation

❒ 5 ❒ 4 ❒ 3 ❒ 2 ❒ 1

28.5) Following the hospital's policy

❒ 5 ❒ 4 ❒ 3 ❒ 2 ❒ 1

29. A 50-year-old woman is diagnosed with supraventricular tachycardia status post radiofrequency ablation. Currently, she is not on any medications. She does not have dyspnea or palpitation, and she is able to do a 1-hour bicycle ride. The patient comes to the hospital because of burning abdominal pain which is aggravated with stress. She is diagnosed with dyspepsia and will be scheduled for EGD. How do the following factors affect your decision to request preoperative chest radiography?

29.1) Avoidance of operation cancellation by surgeons and anesthesiologists

❒ 5 ❒ 4 ❒ 3 ❒ 2 ❒ 1

29.2) Tuberculosis surveillance

❒ 5 ❒ 4 ❒ 3 ❒ 2 ❒ 1

29.3) Prevention of risks or complications during the operation

❒ 5 ❒ 4 ❒ 3 ❒ 2 ❒ 1

29.4) Prevention of prosecution should there be adverse events during the operation

❒ 5 ❒ 4 ❒ 3 ❒ 2 ❒ 1

29.5) Following the hospital's policy

❒ 5 ❒ 4 ❒ 3 ❒ 2 ❒ 1

30. A 35-year-old female patient with a history of pulmonary tuberculosis during the 2 years prior to admission is scheduled for an appendectomy. She has completed a 6-month period of tuberculosis treatment. How do the following factors affect your decision to request preoperative chest radiography?

30.1) Avoidance of operation cancellation by surgeons and anesthesiologists

❒ 5 ❒ 4 ❒ 3 ❒ 2 ❒ 1

30.2) Tuberculosis surveillance

❒ 5 ❒ 4 ❒ 3 ❒ 2 ❒ 1

30.3) Prevention of risks or complications during the operation

❒ 5 ❒ 4 ❒ 3 ❒ 2 ❒ 1

30.4) Prevention of prosecution should there be adverse events during the operation

❒ 5 ❒ 4 ❒ 3 ❒ 2 ❒ 1

30.5) Following the hospital's policy

❒ 5 ❒ 4 ❒ 3 ❒ 2 ❒ 1

31. Do you consider that the current preoperative evaluation guidelines of Siriraj Preanesthetic Clinic (SiPAC) could reduce and prevent complications during the operations?

❒ 5 ❒ 4 ❒ 3 ❒ 2 ❒ 1

32. Do you consider that following the current SiPAC preoperative evaluation guidelines could prevent cancellations of operations by surgeons and anesthesiologists?

❒ 5 ❒ 4 ❒ 3 ❒ 2 ❒ 1

33. Do general medical personnel strictly follow the SiPAC preoperative evaluation guidelines?

❒ 5 ❒ 4 ❒ 3 ❒ 2 ❒ 1

34. Are you concerned about adverse events or complications during an operation if chest radiography for the patient is not available preoperatively?

❒ 5 ❒ 4 ❒ 3 ❒ 2 ❒ 1
