# Supplementary material for: Factors influencing preoperative chest radiography request for elective endoscopic procedures among medical personnel
Source: PLoS One. 2020 Nov 13;15(11):e0242140. doi: 10.1371/journal.pone.0242140 (PMC7665807; doi:10.1371/journal.pone.0242140)
Supplement: Attachment — Submitted filename: plos one review.docx [file pone.0242140.s003.docx]

**Medical Personnel Knowledge and Attitudes About Preoperative Chest Radiography for Elective Endoscopic Procedures**

Pawit Somnuke M.D. Ph.D.^1^; Rachaneekorn Ramlee M.Sc.^2^; Waratchaya Ratanapaiboon M.D.^3^; Passorn Thommaaksorn B.Ns.^1^; Cherdsak Iramaneerat M.D., Ph.D.^4^; Arunotai Siriussawakul M.D.^1,2*^

^1^ Department of Anesthesiology, Faculty of Medicine, Siriraj Hospital, Mahidol University, Bangkok 10700, Thailand

^2^ Integrated Perioperative Geriatric Excellent Research Center, Faculty of Medicine, Siriraj Hospital, Mahidol University, Bangkok 10700, Thailand

^3^ Department of Anesthesiology, Veterans General Hospital, Bangkok 10400, Thailand

^4^ Department of Surgery, Faculty of Medicine, Siriraj Hospital, Mahidol University, Bangkok 10700, Thailand.

***Corresponding Author**

Email address: arunotai.sir@mahidol.ac.th (AS)

**Medical Personnel Knowledge and Attitudes About Preoperative Chest Radiography for Elective Endoscopic Procedures**

**Abstract**

**Background:** Chest radiography is not routinely recommended before elective endoscopies. A high incidence of perioperative chest radiography requests was observed at our institution for tuberculosis screening. This study assessed medical personnel’s knowledge and attitudes about preoperative chest radiography for patients undergoing gastrointestinal endoscopies.

**Methods:** This prospective study recruited 264 participants from different medical specialties. They completed questionnaires exploring their knowledge and attitudes about preoperative chest radiography. A retrospective review was also conducted of 2,030 patients scheduled for elective endoscopic procedures.

**Results:** The questionnaire response rate was 53.79%. The mean scores for knowledge and attitudes were comparable among all medical specialties. Although individuals responsible for ordering chest radiography scored significantly higher on its necessity for pediatric patients than those who were not responsible, they scored lower on the radiographic requirements of patients without underlying diseases. Male respondents expressed concern about radiography requests for patients older than 45 and tuberculosis screening. Correlations between knowledge and attitudes were observed for patient’s age, low-risk surgery and health concerns, and medicolegal and work-policy issues. No correlations were found for tuberculosis. The proportions of respondents with good knowledge and attitudes from all identified correlations were 4.22%–40.85%. While the chart reviews revealed preoperative chest radiography requests were made for 35.52% of patients, only 0.2% had clinical abnormalities.

**Conclusions:** Despite the correlations with patients’ age, low-risk surgery and health concerns, and medicolegal and work-policy issues, the population with good knowledge and attitudes was minimal. Preoperative knowledge and attitudes—especially about tuberculosis—need to be enhanced.

**Key Words:** Chest radiography, knowledge and attitudes, elective surgery, endoscopic procedure, medical personnel

**Introduction**

Preoperative assessments are considered prerequisite for patients undergoing surgical procedures in elective or emergency settings. Adequate tests are helpful in identifying and determining risks, optimizing anesthetic techniques to reduce morbidity and mortality, directing postoperative management, and preventing prosecutions should there be adverse events during the operations [1]. However, preoperative screening can be costly, resource intensive, time-consuming, and uncomfortable for patients. Ideally, the ordering of preprocedural testing should be based on data gathered from patients, comprising their histories, comorbidities, and significant findings from physical examination (ASGE guidelines [2]). Notwithstanding the paramount need for preoperative testing, several studies have concluded that no benefit is provided by routine testing in cases of elective, low-risk, ambulatory surgery for either adult or pediatric patients [3, 4].

Chest radiography is one of the frequent paradigms of preoperative tests covered by recent preoperative guidelines for elective surgery. It is recommended for patients over 60 years of age, especially those with a history of smoking, an ASA physical status of 3 or higher, a respiratory tract infection, signs and symptoms of cardiopulmonary disease, and decompensated heart failure [5–8]. In contrast, chest radiography is not routinely recommended before certain elective surgeries, especially endoscopies, because the incidence of radiographic figures that alters clinical outcomes is as low as 0.1% among all abnormal images [9, 10], and the endoscopic procedures themselves are regarded as low risk, having a cardiac event risk of less than 1%. Previous studies demonstrated that there were insufficient data to determine the benefits of routine laboratory testing before endoscopic procedures; nevertheless, surgeons were inclined to unnecessarily request routine laboratory and preoperative screening tests [6, 11–14]. Barely 1% of the tests from overall patients have been reported to reveal abnormalities that would affect perioperative outcomes [15]. Routine testing in cases of low-risk surgery may result in extra testing, exposure to radiation, surgery cancellation, increased patient anxiety, and additional hospital expenses [4, 5, 16, 17]. Still, a number of physicians express concern about tuberculosis in patients awaiting scheduled surgeries, especially in endemic areas where the prevalence is high, such as in Asian countries. As evidenced by the Global Tuberculosis Report of the World Health Organization 2016, Thailand is among the 14 countries with the highest disease severity [18, 19]. Although there are around 120,000 new cases per annum (equivalent to 171 cases per 100,000 of the Thai population), only 55.3% of the cases are detectable. It is therefore still controversial whether chest radiographic examinations form a useful part of preoperative evaluations and should be no longer considered as mandatory by preoperative guidelines.

In an investigation of the causes of unnecessary testing, Brown et. al. found several influencing factors, such as practice traditions, the belief that other physicians want the tests done, medicolegal concerns, a desire to avoid surgical delays or cancellations, and a lack of awareness of evidence and guidelines [20]. The preoperative knowledge of the majority of medical personnel might not be adequate for the decision-making involved in ordering preoperative tests. Having adequate knowledge and proper attitudes are also pivotal elements for requesting appropriate preoperative investigations. To date, there have not been any studies on the relationships between knowledge and attitudes with the actual practice of preoperative chest radiography. This study was designed to determine the knowledge and attitudes of medical personnel in the residency and fellowship training programs about the importance and necessity of preoperative chest radiography in patients undergoing gastrointestinal endoscopy. Moreover, the rate of preoperative chest x-ray (CXR) requests among outpatients scheduled for endoscopies and the proportion of requested films that involved abnormal chest radiographs were ascertained.

**Materials and Methods**

**Study design and participants**

This prospective clinical study was conducted at a tertiary-care, university-based hospital. The 264 participants were recruited from the residency and fellowship training programs of the Departments of Anesthesiology (80 individuals), Surgery (171), and Gastrointestinal Medicine (13), all under the Faculty of Medicine. The inclusion criteria were medical personnel who agreed to participate in the study.

In addition, an observational retrospective study was conducted by reviewing 2,030 outpatient charts produced January–November 2017. The patients were scheduled for elective endoscopic procedures, which were performed by either surgeons or gastroenterology medical staff under intravenous sedation administered by anesthesiologists. The chest radiography data during the 6 months preceding the patients’ scheduled endoscopic procedures were gathered and analyzed. Patients meeting all of the following criteria were included in the review: aged ≥ 18 years, and outpatients with an appointment for esophagogastroduodenoscopy and/or colonoscopy. Excluded were patients with incomplete medical records, uninterpretable x-ray results (for instance, due to suboptimal inspiration), or no chest-film interpretation by a radiologist. The protocol for the study was approved by the Siriraj Institutional Review Board, Faculty of Medicine, Siriraj Hospital, Mahidol University, Bangkok, Thailand.

**Data collection**

The questionnaire was created by 5 specialists from different fields of expertise (namely, anesthesiology, surgery, and psychology) to assure content validity, accuracy, and appropriateness of the questions. The questionnaire aimed to determine the factors involved in decision-making by the residents and fellows with regard to their preoperative chest radiography requests. The Index of Consistency score of the questionnaire was 0.91. The questionnaire was categorized into 3 main sections. Part 1 dealt with general information on the participants, comprising their age, sex, education, the number of gastrointestinal endoscopic performances during the preceding 3 months, responsibility for ordering preoperative CXRs, and participation in preoperative CXR evaluation courses. Part 2 focused on the participants’ basic knowledge of the necessity and importance of preprocedural or preoperative chest radiography, drawing upon information contained in the guidelines of the American Society of Anesthesiologists and the National Institute for Clinical Excellence, UK. Finally, Part 3 addressed the participants’ attitudes towards preprocedural or preoperative requests for chest radiography. Each item under the basic knowledge category was a yes–no question, while the items under the attitude category were rated in Likert-scale fashion, such as “extremely important” (5), “very important” (4), “moderately important” (3), “slightly important” (2), and “very slightly important” (1). The knowledge test consisted of 18 questions; 7 were about surgery in ambulatory settings, whereas the others dealt with general, preoperative, CXR indications, and knowledge of tuberculosis. The attitude test consisted of 11 questions. The questionnaires were delivered to the residents and fellows in closed envelopes. A research assistant requested the participants to return the questionnaires within 2 weeks. After the participants had completed the questionnaires, the scores for the items were calculated and summarized.

**Statistical analysis**

The demographic characteristics of the respondents were utilized as background independent variables and described by mean, percentage, and standard deviation. In addition, the scores from the knowledge and attitude sections of the questionnaire were summarized and compared between respondent groups by mean, percentage, and standard deviation. Statistical significances were determined by chi-squared tests. A p-value of less than 0.05 was regarded as statistically significant. Spearman’s rank correlation was used to study the association between knowledge and attitudes.

The sample size of the retrospective patient chart review was calculated in a similar fashion to a previous study [21]. We assumed that the frequency of the preprocedural chest radiography requests for the patients in the endoscopic division was 78% (p = 0.78), with a confidence level of 95% and an allowable error of 2% (d = 0.02). The calculated sample size was 1,648. However, as we included an additional 15% of the sample size to compensate for incomplete or missing medical record data, the final sample size was 2,030.

The preoperative chest radiography statuses of the patients were registered, and the abnormal preoperative chest radiography results were recorded and reported as percentages. All demographic data were analyzed using descriptive statistics. The data with a normal distribution were analyzed and reported as percentage, mean, and standard deviation. SPSS Statistics for Windows, version 18.0 (SPSS Inc., Chicago, Ill., USA) was used for the analyses.

**Results**

Although 264 medical personnel were recruited to the study, only 142 (53.79%) returned completed questionnaires. Of those respondents, 54 were from the Department of Anesthesiology, 77 from Surgery, and 11 from Gastrointestinal Medicine. Surgeons represented the major subpopulation group (54.22%), followed by anesthesiologists (38.03%) and gastroenterologists (7.75%). Table 1 details the baseline characteristics of the participants (age, gender, position in training programs, year of training, preoperative CXR course attendance, being a key individual to order preoperative CXRs, and GI endoscopic performance during the preceding 3 months).

The average participant age was approximately 29 years. There were significant mean age differences between the 3 groups of medical professions (anesthesiologists, surgeons, and gastroenterologists), with the highest mean age being for gastroenterologists (31.27 ± 1.74 years), followed by surgeons (29.17 ± 2.35) and anesthesiologists (28.24 ± 1.44). The overall proportions of male and female participants were 47.2% and 52.8%, respectively. Although the large majority of the anesthesiologists (85.2%) were female, the surgeons were predominantly male (68.8%). All members of the anesthesiology group were 1^st^–3^rd^ year residents-in-training, while all gastroenterologists were in the 1^st^ or 2^nd^ year of their clinical fellowship training program. As to the surgery group, there were mixed populations: 10.4% were in their 4^th^ or 5^th^ training year and were thus considered as fellows, but the vast majority were residents. About half of the participants in all groups had attended a preoperative CXR course. Although more than 90% of the members of the surgery and gastroenterology groups were responsible for ordering preoperative CXRs, less than half of the members of the anesthesiology group had this role. Finally, the majority of the anesthesiologists and surgeons had performed up to 10 GI endoscopies during the preceding 3 months; by contrast, most of the gastroenterologists had conducted up to 20 cases (Table 1).

The knowledge and attitudes about preoperative chest radiography of all of the participants were subsequently assessed by using a questionnaire (Appendix) composed of 2 question sets (one dealing with knowledge, and the other with attitudes). The original version of the questionnaire had been designed and validated by experts at the Faculty of Medicine, Siriraj Hospital, Mahidol University. The questionnaire had a total of 34 questions: 18 yes–no questions in the knowledge section, and another 8 in the attitudes section. Certain questions that were related to elective and gastrointestinal endoscopic procedures were subsequently selected (9 from the knowledge set, and 4 from the attitude set); the 4 attitude-related questions were then further subdivided into 17 questions. That meant that the final version of the questionnaire had 9 knowledge- and 17 attitude-related questions. The knowledge questions were categorized into 3 main topics. The first, “patient’s age”, dealt with the appropriate age for preoperative CXRs. The second class of questions, “low-risk surgery and health concerns”, examined the need for CXRs in cases of low-risk surgical procedures or low-risk patient health conditions, such as heart disease and asthma. The final category, “TB awareness”, related to the need for preoperative CXRs in particular situations, such as patients with a TB history or as part of TB surveillance activities. Correct answers to the knowledge questions were scored 1, whereas incorrect answers were scored 0. The attitude questions were categorized into 4 main topics: patient’s age, low-risk surgery and health concerns, medicolegal and work-policy issues, and TB awareness. The items under the attitude category were rated in Likert-scale fashion, such as “extremely important” (5), “very important” (4), “moderately important” (3), “slightly important” (2), and “very slightly important” (1). Therefore, good attitudes were regarded as scores 1–3, and poor attitudes scored 4–5.

With regard to the 3 questions in the patient’s-age category, there were no statistically significant differences in the mean scores of the 3 work groups. However, in the case of question number 9 (Q9), which asked whether all patients of any age who are undergoing surgery should have preoperative CXRs, the gastroenterologists had a significantly higher proportion of correct answers (36.4%) than the other two work groups. They were followed by surgeons, with 29.9% correct, and anesthesiologists (7.4%). Another noticeable difference was observed with Q14, which asked whether a patient with non-active asthma requires preoperative chest radiography. For that question, surgeons had a statistically significantly higher proportion of correct answers (79.2%) than anesthesiologists (57.4%) and gastroenterologists (36.4%). Turning to the 3 questions relating to low-risk surgery and health concerns, the mean scores for the 3 groups were comparable. Finally, the mean scores for the responses to the questions under the category concerning tuberculosis did not demonstrate any differences between the groups (Table 2).

As to the attitude questions, there were no statistically significant differences between the 3 groups of participants when analyzed under the categories of patient’s age, low-risk surgery and health concerns, and medicolegal and work-policy issues. Even though the mean scores for attitudes towards tuberculosis did not differ among the 3 groups, there were still statistically significant differences in attitudes when focusing on certain topics, such as the importance of a preprocedural CXR in a patient with a history of tuberculosis (Q27.1), and whether a CXR should be ordered for a patient who had completed tuberculosis treatment in order to avoid case cancellation (Q30.1). Gastroenterologists seemed to be the most concerned, indicated by having the highest attitude scores (followed by anesthesiologists and surgeons) about performing chest radiography in previously infected patients. On the other hand, surgeons appeared to be the most worried (followed by anesthesiologists and gastroenterologists) about a case cancellation if a preoperative CXR was not conducted (Table 3).

The respondents were further classified according to sex; position in their training programs (resident or fellow); department (Anesthesiology, Surgery, or Gastrointestinal Medicine); prior preoperative CXR course attendance (yes–no); and being responsible for ordering preoperative CXRs (yes–no). They were subsequently analyzed by using the same sets of knowledge and attitude questions. Instead of calculating mean scores, the percentage of respondents with correct answers for the knowledge questions (Table 4) and the proportion of respondents with poor attitudes (scores 4 and 5 for the attitude-related questions) were obtained for analysis (Table 5). In concordance with the previous results of Table 2, gastroenterologists and surgeons significantly scored the highest among the members of the department subgroup for Q9 and Q14 of the knowledge questions, respectively. The percentage of respondents with correct answers for Q12 (asking whether preprocedural CXR is important for all pediatric patients) was significantly higher for individuals who were key to preoperative-CXR ordering than for those who were not. In contrast, the percentage of correct answers for respondents who were not responsible for CXR ordering was significantly higher when assessed for Q20 (involving the necessity of preoperative CXR for esophagogastroduodenoscopy) than the corresponding figure for individuals who were key to preoperative-CXR ordering. No significant differences were observed among other subgroups. The subgroups relating to sex, department, and being key individuals for CXR ordering demonstrated significant differences in the proportion of respondents with poor attitudes. In the case of Q27.2 (under the attitude topic of low-risk surgery and health concerns), all anesthesiologists and gastroenterologists showed poor attitudes toward the importance of CXR before an endoscopic procedure in a patient with heart disease, whereas a poor attitude was seen in around 90% of the surgeons. As to Q29.1 (focusing on the requirement for CXR before esophagogastroduodenoscopy in a patient with supraventricular tachycardia), surgeons were the leading respondents with poor attitudes (61.0%), followed by anesthesiologists (44.4%) and gastroenterologists (27.3%), based on a fear of case cancellation. With regards to TB awareness, all anesthesiologists and gastroenterologists had poor attitudes toward the importance of a pre-endoscopic CXR in a patient with a history of pulmonary TB (Q27.1), as opposed to a significantly lower percentage of poor attitudes among surgeons (89.6%). Similarly, Q30.1 (dealing with the need for preoperative CXR for appendectomy in a patient with a history with pulmonary TB to prevent a case cancellation) revealed that surgeons were the leading population with the highest percentage of poor attitudes (81.8%), followed by anesthesiologists (64.8%) and gastroenterologists (45.5%). Male respondents had a significantly higher percentage of poor attitudes toward TB, due to their willingness to avoid operation cancellation (Q30.1) and prevent risks and complications during the operation (Q30.3; Table 5). Additionally, when assessed by Q27.6, male respondents (61.2%) and the ones who were key individuals for CXR ordering (55.7%) had the higher propensity to request preoperative CXRs, and regardless of the appropriate age of the patients.

The correlations between the knowledge and attitudes of the 3 groups were calculated by Spearman’s rank correlation test. Albeit quite low, significant correlations between the overall knowledge and attitude scores of all of the respondents were observed when the knowledge topics were paired with the attitude questions, and when the attitude topics were paired with the attitude questions. Significant correlations between knowledge and attitudes were achieved on topics such as patient’s age, low-risk surgery, health concerns, and medicolegal and work-policy issues. There was a negative correlation (correlation coefficient r, -0.201) between knowledge and attitudes relating to the topic of patient’s age. However, positive correlations were observed between knowledge and attitudes under the topic of low-risk surgery and health concerns (r, 0.311), and between the knowledge questions related to low-risk surgery and health concerns and the attitude questions on medicolegal and work-policy issues (r, 0.257). In addition, the pairing between 2 attitude topics—low-risk surgery and health concerns, and medicolegal and work-policy issues—showed a significantly positive correlation (r, 0.335). No correlations were observed for TB awareness (Table 6).

To explore the aspects of knowledge and attitudes that need improvement, the proportions of respondents with good knowledge (“Good K”) and good attitudes (“Good A”) were calculated (Table 7). The respondents who scored higher than 60% for overall knowledge and attitude assessment under each category were considered as having good knowledge or good attitudes (1 out of 1 for a set comprised of a single question; 2 out of 3 for a set of 3 questions; 3 out of 4 for a set of 4 questions; and 5 out of 8 for a set of 8 questions). Most of the medical personnel had good preoperative knowledge of patients’ age-appropriateness for CXR requests (85.21%), whether CXRs were necessary for elective surgeries, and patients with medical conditions (74.64%). However, less than half of the respondents possessed good knowledge of TB (34.51%). Only 50% of all respondents presented good attitudes to patients’ age appropriateness, while 30.28% and 42.95% of the population had good attitudes toward low-risk surgery and health concerns, and to medicolegal and work-policy issues, respectively. The proportion of individuals with good attitudes about TB awareness was as low as 7.04%. The majority of respondents—determined by the correlations between knowledge and attitudes about low-risk surgery and health concerns, and between knowledge of low-risk surgery and health concerns and attitudes to medicolegal and work-policy issues—were the population with good knowledge but poor attitudes (Good K, Poor A; 70.42% and 45.77%, respectively). Roughly 40% of the respondents who were assessed for correlation under the topic of age appropriateness for CXR were the ones with good knowledge and good attitudes (Good K, Good A; 40.85%) and good knowledge but poor attitudes (Good K, Poor A; 44.37%). In contrast, the respondents with poor knowledge and poor attitudes (Poor K, Poor A) were the majority of the population assessed under the correlation between knowledge and attitudes about TB awareness (47.18%) and the correlation between attitudes toward low-risk surgery and health and medicolegal and work-policy issues (53.52%).

**Discussion**

It is well documented that preoperative chest radiography, especially in ambulatory settings, might not be essential. This is because only 12% of patients are found to have abnormal CXR findings and, interestingly, as few as 0.03% need further investigation and treatment [9]. Data related to 2,030 patients was collected by our retrospective patient chart review of endoscopic cases scheduled in outpatient settings at Siriraj Hospital, Mahidol University, Thailand, between January and November 2017. Of those, 721 cases (35.52%) had CXRs during the 6 months preceding their operations. Abnormal radiographic findings were found by radiologists in 396 cases (19.51%). As 87 of those cases were subsequently determined to have non-significant findings (for instance, cardiomegaly, calcified aortas, and inactive pulmonary infiltration), only the 309 remaining cases (15.22%) exhibited true abnormalities. Clinical abnormalities (chronic obstructive pulmonary disease, active tuberculosis, and lung nodules suspected for malignancy) were observed in 5 cases (0.25%), and only 4 patients (0.20%) needed further investigation and medical consultation. Our retrospective data indicated that that there was an over-investigation of the preprocedural chest radiography (35.52%, compared with 12% as previously reported for the actual incidence of preoperative abnormal films [9]). In addition, proper guidelines for the preoperative preparation of elective ambulatory cases are yet to be established. For these reasons, it would be invaluable to clarify the rationale for preoperative CXR requests. Our study focused on a diverse population of medical professions—anesthesiologists, surgeons, and gastroenterologists—who had experience with patient evaluations and the requesting of tests preoperatively. We assessed their knowledge and attitudes by analyzing their scores for a validated questionnaire. Initially, it was assumed that knowledge would correlate well with attitudes about preoperative CXR requests; in other words, that having a good knowledge would result in appropriate attitudes toward CXR requests for ambulatory cases.

Most of the surgeons and gastroenterologists were responsible for ordering preoperative CXRs, which might be why they scored higher than anesthesiologists on Q9 (whether all patients undergoing surgery needed preoperative CXRs). Also, surgeons were striking leaders in terms of the percentage of correct answers to Q14 (whether a non-active asthmatic patient requires a preoperative CXR). The medical personnel who had been specially trained in medicine had had a greater chance of encountering tuberculosis patients who, typically, do not show clinical symptoms yet are still contagious. Therefore, the reason why the gastroenterologists exhibited the lowest percentage of staff with correct answers to the questions related to the prevalence and specificity of CXR for tuberculosis (Q24 and Q26) might be because they are strongly concerned about patients at risk, as evidenced by their high attitude scores for the tuberculosis-related questions (Q27.1, Q28.2, and Q30.2), rather than because of an awareness of the possible complications during operations (Q30.3), or concern about case cancellations (Q30.1) or prosecution (Q30.4). However, the number of gastroenterologists was much less than the numbers for the staff in the 2 other groups, which is consistent with the limited number of training positions in gastroenterology. Therefore, better results and statistical significance might be achieved with an increased number of gastrointestinal-medicine respondents. The results of our analysis revealed no significant differences in the mean knowledge and attitudes of all 3 groups of respondents. Nevertheless, the study revealed particular aspects (such as the appropriate age of patients scheduled for surgery that require preoperative CXRs, and the importance of tuberculosis for preoperative-CXR ordering) that need to be highlighted among all medical professions.

After regrouping the respondents, we still found that having been in a training program as a resident or as an individual responsible for CXR ordering might have been beneficial in excluding non-specific pain at the epigastrium (Q20) from the items needing a preoperative CXR request. Male respondents expressed more concern about CXR requests before the procedures when it came to the matter of the appropriate age of patients who were undergoing surgeries (Q27.6), past histories of tuberculosis for fear of case cancellation (Q30.1), and risks and complication during the procedures (Q30.3). This might be because of a better decision-making ability of female respondents, rather than knowledge, since the female respondents had comparable overall knowledge scores to the males. The individuals who had attended a preoperative CXR course and had experienced being key individuals for CXR ordering did not express significantly different knowledge or attitudes than those personnel without course attendance or without experience of CXR ordering. In other words, greater experience—either via a preoperative CXR course or being the person in charge of CXR ordering—might not be reflected in better knowledge and attitude about preoperative CXRs.

It was initially assumed that a negative correlation between knowledge and attitude about patients’ age appropriateness for preoperative CXRs would mean a reverse correlation between knowledge and attitudes; thus, high knowledge scores (Good K) would be seen with low attitude scores (Good A). We further considered that positive correlations from other knowledge-attitude pairings involving the low-risk surgery and health concerns and the medicolegal and work-policy issues might indicate an inclination to request CXRs for patients who have medical conditions that present a risk of prosecution, and/or to prevent possible violations of work policies relating to requests for CXRs, regardless of the need to include CXRs in preoperative investigations. Moreover, we speculated that a positive correlation between 2 different aspects of attitude—low-risk surgery and health concerns, versus medicolegal and work-policy issues—might also emphasize that the high rate of CXR ordering is because of concerns about risks and complications, medical litigation, and/or ignoring workplace policies. When assessments were made of the subpopulation under each topic of interest, the results somewhat corresponded with our hypotheses. The majority of respondents determined by patients’ age appropriateness had either good knowledge and good attitudes, or good knowledge and poor attitudes, with only a small number of respondents having poor knowledge and poor attitudes. When determined by low-risk surgery and health concerns and medicolegal and work-policy issues, the majority of respondents belonged to the good knowledge–poor attitude population, while the ones with good knowledge and good attitudes were minimal. Given those results, it could be interpreted that patients’ age appropriateness might be the easiest topic to enhance good knowledge and good attitudes among medical personnel. However, it is important to raise awareness of the need to develop and consolidate every aspect of preoperative knowledge and attitudes among all medical personnel, especially with regard to tuberculosis, in order to sharpen their judgement and decision-making skills and thus ensure proper requests are made for preoperative chest radiography. Future research could determine the actual practices of medical personnel relating to preoperative-CXR ordering when their level of knowledge and attitudes are properly enhanced in order to avoid excessive and costly preoperative-CXR requests.

**Acknowledgments**

This study was facilitated by the Integrated Perioperative Geriatric Excellent Research Center, Faculty of Medicine, Siriraj Hospital, Mahidol University, Bangkok, Thailand. The authors are grateful to Assist. Prof. Dr. Chulaluk Komoltri of the faculty’s Clinical Epidemiology Unit for the statistical analyses.

**References**

1.	Feely, M.A., et al., *Preoperative testing before noncardiac surgery: guidelines and recommendations.* Am Fam Physician, 2013. 87(6): p. 414–8.

2.	Committee, A.S.o.P., et al., *Routine laboratory testing before endoscopic procedures.* Gastrointest Endosc, 2014. 80(1): p. 28–33.

3.	Benarroch-Gampel, J., et al., *Preoperative laboratory testing in patients undergoing elective, low-risk ambulatory surgery.* Ann Surg, 2012. 256(3): p. 518–28.

4.	Chung, F., et al., *Elimination of preoperative testing in ambulatory surgery.* Anesth Analg, 2009. 108(2): p. 467–75.

5.	O'Neill, F., et al., *Routine preoperative tests for elective surgery: summary of updated NICE guidance.* BMJ, 2016. 354: p. i3292.

6.	Smetana, G.W. and D.S. Macpherson, *The case against routine preoperative laboratory testing.* Med Clin North Am, 2003. 87(1): p. 7–40.

7.	Committee on, S., et al., *Practice advisory for preanesthesia evaluation: an updated report by the American Society of Anesthesiologists Task Force on Preanesthesia Evaluation.* Anesthesiology, 2012. 116(3): p. 522–38.

8.	Richman, D.C., *Ambulatory surgery: how much testing do we need?* Anesthesiol Clin, 2010. 28(2): p. 185–97.

9.	Lim, E.H. and E.H. Liu, *The usefulness of routine preoperative chest X-rays and ECGs: a prospective audit.* Singapore Med J, 2003. 44(7): p. 340–3.

10.	Silvestri, L., et al., *Usefulness of routine pre-operative chest radiography for anaesthetic management: a prospective multicentre pilot study.* Eur J Anaesthesiol, 1999. 16(11): p. 749–60.

11.	Dzankic, S., et al., *The prevalence and predictive value of abnormal preoperative laboratory tests in elderly surgical patients.* Anesth Analg, 2001. 93(2): p. 301–8, 2nd contents page.

12.	Haug, R.H. and R.L. Reifeis, *A prospective evaluation of the value of preoperative laboratory testing for office anesthesia and sedation.* J Oral Maxillofac Surg, 1999. 57(1): p. 16–20; discussion 21–2.

13.	Pasternak, L.R., *Preoperative laboratory testing: general issues and considerations.* Anesthesiol Clin North Am, 2004. 22(1): p. 13–25.

14.	Robinson, T.N., et al., *Routine preoperative laboratory analyses are unnecessary before elective laparoscopic cholecystectomy.* Surg Endosc, 2003. 17(3): p. 438–41.

15.	Kaplan, E.B., et al., *The usefulness of preoperative laboratory screening.* JAMA, 1985. 253(24): p. 3576–81.

16.	Fritsch, G., et al., *Abnormal pre-operative tests, pathologic findings of medical history, and their predictive value for perioperative complications.* Acta Anaesthesiol Scand, 2012. 56(3): p. 339–50.

17.	Schein, O.D., et al., *The value of routine preoperative medical testing before cataract surgery. Study of Medical Testing for Cataract Surgery.* N Engl J Med, 2000. 342(3): p. 168–75.

18.	MacNeil, A., et al., *Global Epidemiology of Tuberculosis and Progress Toward Achieving Global Targets—2017.* MMWR Morb Mortal Wkly Rep. 68(11): p. 263–266.

19.	World Health Organization. Global tuberculosis report 2016. Geneva, Switzerland: World Health Organization; 2016. https://www.who.int/tb/publications/global_report/en/

20.	Brown, S.R. and J. Brown, *Why do physicians order unnecessary preoperative tests? A qualitative study.* Fam Med, 2011. 43(5): p. 338–43.

21.	Chinachot, T., et al., *Incidence, severity and factors lead to abnormal preoperative blood chemistry, CBC, Chest X-ray and EKG in routine preoperative investigation.* Thai Journal of Anesthesiology, 2016. 42(2): p. 69–87.

**Appendix**

**Questionnaire for assessment of knowledge and attitudes of medical personnel about preprocedural or preoperative requests for chest radiography**

This questionnaire consists of 3 parts. Please answer all of the following questions according to your actual opinion. Your answers are valuable for our research on the knowledge, attitudes, and actual practices of medical personnel on preprocedural or preoperative requests for chest radiography. This research may assist with the development of appropriate guidelines for preprocedural or preoperative chest radiography requests at Siriraj Hospital. Please feel assured that your answers will be kept confidential, and they will not be individually analyzed.

Our research team recognizes that that this questionnaire is relatively long and therefore may take some time to complete. However, we greatly hope to get your cooperation.

Thank you very much for giving your time and making the effort to contribute to this research for the benefit of society.

**Part1. General information**

1. Age: ……………. years
2. Gender

❒ 1. Female	❒ 2. Male

3. Position

❒ 1. Resident	❒ 2. Fellow

4. Department

❒ 1. Anesthesiology	❒ 2. Surgery	❒ 3. Gastrointestinal Medicine

5. Year of training

❒ 1. 1^st^ year	❒ 2. 2^nd^ year	❒ 3. 3^rd^ year

❒ 4. 4^th^ year	❒ 5. 5^th^ year

6. Have you ever attended any courses on the preoperative chest radiography?

❒ 0. No	❒ 1. Yes

7. Are you the person who decides to make requests for preoperative chest radiography?

❒ 0. No	❒ 1. Yes

8. How many patients have you examined and proceeded to endoscopic procedures during the past 3 months?

❒ 1. 0-10	❒ 2. 10-20	❒ 3. 20-30

❒ 4. 30-40	❒ 5. > 40

**Part 2. Basic knowledge about preprocedural or preoperative chest radiography**

**The following questions are designed to assess your knowledge. Your answers will not affect your current work. Please select your answer and mark** ✓ **in the** ❒**.**

9. All patients who are undergoing surgery should have preprocedural chest radiography.

❒ 0. No	❒ 1. Yes

10. All patients aged over 45 years should have preprocedural chest radiography.

❒ 0. No	❒ 1. Yes

11. All patients scheduled for major surgeries (for example, kidney transplantation) should have preprocedural chest radiography.

❒ 0. No	❒ 1. Yes

12. All pediatric patients should have preprocedural chest radiography.

❒ 0. No	❒ 1. Yes

13. A 39-year-old male patient with atrial fibrillation is scheduled for hernioplasty. He should have chest radiography before the operation.

❒ 0. No	❒ 1. Yes

14. A 35-year-old woman with asthma is scheduled for colonoscopy. She has not developed active asthma for 1 year and therefore does not require any bronchodilators. Chest radiography should be required before the procedure.

❒ 0. No	❒ 1. Yes

15. A 20-year-old man has rheumatic heart disease. He does not have dyspnea and can walk up 2 flights of stairs. The patient is scheduled for mitral valve replacement. Chest radiography should be required before the operation.

❒ 0. No	❒ 1. Yes

16. A 50-year-old man has chronic obstructive pulmonary disease (COPD). He stopped smoking 10 years ago, and he has not had any COPD exacerbation that required hospitalization during the last 2 years. He is scheduled for transurethral resection of the prostate (TURP). Chest radiography should be required before the operation.

❒ 0. No	❒ 1. Yes

17. A 50-year-old female patient with chronic kidney disease (CKD) requiring hemodialysis 2 times/week is scheduled for cataract surgery. She does not have a history of pulmonary edema. Chest radiography should be required before the operation.

❒ 0. No	❒ 1. Yes

18. A 30-year-old woman with no underlying diseases is scheduled for thyroidectomy. Chest radiography should be required before the operation.

❒ 0. No	❒ 1. Yes

19. A 70-year-old man with ischemic heart disease is scheduled for pylorus-preserving pancreaticoduodenectomy. He does not have chest pain. However, he has to take a rest after walking up 1 flight of stairs. Chest radiography should be required before the operation.

❒ 0. No	❒ 1. Yes

20. A 20-year-old woman with no underlying diseases is complaining about off and on pain at the epigastrium. She is scheduled for esophagogastroduodenoscopy (EGD). Chest radiography should be required before the procedure.

❒ 0. No	❒ 1. Yes

21. A 35-year-old man with esophageal cancer is scheduled for esophagectomy with a gastric pull-up. Chest radiography should be required before the operation.

❒ 0. No	❒ 1. Yes

22. A 30-year-old woman with acoustic neuroma is scheduled for craniotomy with tumor removal. Chest radiography should be required before the operation.

❒ 0. No	❒ 1. Yes

23. A 6**-**hour term male neonate, post conceptual age (PCA) 37 weeks, is diagnosed with gastroschisis. He is scheduled for primary closure. Chest radiography should be required before the operation.

❒ 0. No	❒ 1. Yes

24. The Central region of Thailand is where tuberculosis is most prevalent.

❒ 0. No	❒ 1. Yes

25. During 2017, the incidence of tuberculosis in Thailand was 172 per 100,000 people.

❒ 0. No	❒ 1. Yes

26. Chest radiography is highly specific for tuberculosis.

❒ 0. No	❒ 1. Yes

**Answer key for the knowledge questions**

9: No. 10: No. 11: No. 12: No. 13: No. 14: No. 15: Yes. 16: Yes. 17: No. 18: Yes. 19: Yes.

20: No. 21: Yes. 22: Yes. 23: No. 24: No. 25: Yes. 26: No.

**Part 3. Attitudes about preprocedural or preoperative requests for chest radiography**

**The following questions allow assessment of your opinions on preprocedural or preoperative requests for chest radiography. Please select your answer and mark** ✓ **in the** ❒**.**

**The answers are graded according to the level of importance, namely, extremely important (5), very important (4), moderately important (3), slightly important (2), and very slightly important (1).**

**Please answer all of the questions.**

27. How important is it to chest radiography requests to consider the following factors before an operation or endoscopic procedure?

27.1)	History of pulmonary tuberculosis

❒ 5	❒ 4	❒ 3	❒ 2	❒ 1

27.2)	History of heart disease

❒ 5	❒ 4	❒ 3	❒ 2	❒ 1

27.3)	History of chronic obstructive pulmonary disease (COPD)

❒ 5	❒ 4	❒ 3	❒ 2	❒ 1

27.4)	History of upper respiratory tract infection

❒ 5	❒ 4	❒ 3	❒ 2	❒ 1

27.5)	Smoking

❒ 5	❒ 4	❒ 3	❒ 2	❒ 1

27.6)	Healthy patient older than 45 years with no underlying diseases

❒ 5	❒ 4	❒ 3	❒ 2	❒ 1

28. A 40-year-old male patient with hypertension is diagnosed with acute appendicitis and will be scheduled for appendectomy. He can walk up 2 flights of stairs. He does not have a previous history of chronic cough, chest pain or weakness of extremities. How do the following factors affect your decision to request preoperative chest radiography?

28.1)	Avoidance of operation cancellation by surgeons and anesthesiologists

❒ 5	❒ 4	❒ 3	❒ 2	❒ 1

28.2)	Tuberculosis surveillance

❒ 5	❒ 4	❒ 3	❒ 2	❒ 1

28.3)	Prevention of risks or complications during the operation

❒ 5	❒ 4	❒ 3	❒ 2	❒ 1

28.4)	Prevention of prosecution should there be adverse events during the operation

❒ 5	❒ 4	❒ 3	❒ 2	❒ 1

28.5)	Following the hospital’s policy

❒ 5	❒ 4	❒ 3	❒ 2	❒ 1

29. A 50-year-old woman is diagnosed with supraventricular tachycardia status post radiofrequency ablation. Currently, she is not on any medications. She does not have dyspnea or palpitation, and she is able to do a 1-hour bicycle ride. The patient comes to the hospital because of burning abdominal pain which is aggravated with stress. She is diagnosed with dyspepsia and will be scheduled for EGD. How do the following factors affect your decision to request preoperative chest radiography?

29.1)	Avoidance of operation cancellation by surgeons and anesthesiologists

❒ 5	❒ 4	❒ 3	❒ 2	❒ 1

29.2)	Tuberculosis surveillance

❒ 5	❒ 4	❒ 3	❒ 2	❒ 1

29.3)	Prevention of risks or complications during the operation

❒ 5	❒ 4	❒ 3	❒ 2	❒ 1

29.4)	Prevention of prosecution should there be adverse events during the operation

❒ 5	❒ 4	❒ 3	❒ 2	❒ 1

29.5)	Following the hospital’s policy

❒ 5	❒ 4	❒ 3	❒ 2	❒ 1

30. A 35-year-old female patient with a history of pulmonary tuberculosis during the 2 years prior to admission is scheduled for an appendectomy. She has completed a 6-month period of tuberculosis treatment. How do the following factors affect your decision to request preoperative chest radiography?

30.1)	Avoidance of operation cancellation by surgeons and anesthesiologists

❒ 5	❒ 4	❒ 3	❒ 2	❒ 1

30.2)	Tuberculosis surveillance

❒ 5	❒ 4	❒ 3	❒ 2	❒ 1

30.3)	Prevention of risks or complications during the operation

❒ 5	❒ 4	❒ 3	❒ 2	❒ 1

30.4)	Prevention of prosecution should there be adverse events during the operation

❒ 5	❒ 4	❒ 3	❒ 2	❒ 1

30.5)	Following the hospital’s policy

❒ 5	❒ 4	❒ 3	❒ 2	❒ 1

31. Do you consider that the current preoperative evaluation guidelines of Siriraj Preanesthetic Clinic (SiPAC) could reduce and prevent complications during the operations?

❒ 5	❒ 4	❒ 3	❒ 2	❒ 1

32. Do you consider that following the current SiPAC preoperative evaluation guidelines could prevent cancellations of operations by surgeons and anesthesiologists?

❒ 5	❒ 4	❒ 3	❒ 2	❒ 1

33. Do general medical personnel strictly follow the SiPAC preoperative evaluation guidelines?

❒ 5	❒ 4	❒ 3	❒ 2	❒ 1

34. Are you concerned about adverse events or complications during an operation if chest radiography for the patient is not available preoperatively?

❒ 5	❒ 4	❒ 3	❒ 2	❒ 1

**Supporting information**

S1 File. Raw data of Knowledge and Attitudes About Preoperative Chest Radiography (XLSX)

**Table 1.** Demographic data of the questionnaire respondents

| **Variable** | **n (%)** | | | | **p-value** |
| --- | --- | --- | --- | --- | --- |
|  | **Anesthesiologists**  **(n = 54)** | **Surgeons**  **(n = 77)** | **Gastroenterologists**  **(n = 11)** | **Total** |  |
| **Age (**Years; mean ± SD) | 28.24 ± 1.44 | 29.17 ± 2.35 | 31.27 ± 1.74 | 28.98 ± 2.15 | < 0.001* |
| **Gender** |  |  |  |  |  |
| Female | 46 (85.2) | 24 (31.2) | 5 (45.5) | 75 (52.8) | < 0.001* |
| Male | 8 (14.8) | 53 (68.8) | 6 (54.5) | 67 (47.2) |  |
| **Position** |  |  |  |  |  |
| Resident | 54 (100) | 69 (89.6) | 0 | 123 (86.6) | < 0.001* |
| Fellow | 0 | 8 (10.4) | 11 (100) | 19 (13.4) |  |
| **Year of training** |  |  |  |  |  |
| 1st | 16 (29.6) | 21 (27.3) | 5 (45.5) | 42 (29.6) | 0.947 |
| 2nd | 17 (31.5) | 23 (29.9) | 6 (54.5) | 46 (32.4) |  |
| 3rd | 21 (38.9) | 18 (23.4) | 0 | 39 (27.5) |  |
| 4th | 0 | 11 (14.3) | 0 | 11 (7.7) |  |
| 5th | 0 | 4 (5.2) | 0 | 4 (2.8) |  |
| **Preoperative CXR course attendance** |  |  |  |  |  |
| Yes | 26 (48.1) | 36 (46.8) | 5 (45.5) | 68 (47.9) | 0.888 |
| No | 28 (51.9) | 41 (53.2) | 6 (54.5) | 74 (52.1) |  |
| **Key individual for ordering preoperative CXRs** |  |  |  |  |  |
| Yes | 24 (44.4) | 71 (92.2) | 11 (100) | 106 (74.6) | < 0.001* |
| No | 30 (55.6) | 6 (7.8) | 0 | 36 (25.4) |  |
| **GI endoscopic performance in the preceding 3 months** |  |  |  |  |  |
| 0–10 | 42 (77.8) | 50 (64.9) | 1 (9.1) | 93 (65.5) | 0.069 |
| 10–20 | 4 (7.4) | 16 (20.8) | 5 (45.5) | 25 (17.6) |  |
| 20–30 | 2 (3.7) | 8 (10.4) | 3 (27.3) | 13 (9.2) |  |
| 30–40 | 1 (1.9) | 1 (1.3) | 1 (9.1) | 3 (2.1) |  |
| > 40 | 5 (9.3) | 2 (2.6) | 1 (9.1) | 8 (142) |  |

*Significant at p < 0.05

Abbreviations: CXR, Chest x-ray; GI, Gastrointestinal; SD, standard deviation

**Table 2.** The respondents’ scores for the chest x-ray knowledge questions

| **Factors affecting physician’s decision regarding preoperative CXRs** | **Respondents with correct answers: n (%)** | | | **p-value** |
| --- | --- | --- | --- | --- |
|  | **Anesthesiologists**  **(n = 54)** | **Surgeons**  **(n = 77)** | **Gastroenterologists**  **(n = 11)** |  |
| **Patient’s age** |  |  |  |  |
| Q9 Every patient | 4 (7.4) | 23 (29.9) | 4 (36.4) | **0.004*** |
| Q10 Over 45 years old | 48 (88.9) | 68 (88.3) | 10 (90.9) | 0.967 |
| Q12 All pediatric patients | 50 (92.6) | 69 (89.6) | 9 (81.8) | 0.536 |
| Mean score ± SD | 0.63 ± 0.15 | 0.69 ± 0.20 | 0.70 ± 0.23 | 0.161 |
| **Low-risk surgery and health concerns** |  |  |  |  |
| Q14 Colonoscopy in non-active asthma | 31 (57.4) | 61 (79.2) | 4 (36.4) | **0.002*** |
| Q17 Cataract surgery in CKD requiring hemodialysis | 41 (75.9) | 44 (57.1) | 6 (54.5) | 0.069 |
| Q20 EGD in no underlying disease | 38 (70.4) | 58 (75.3) | 6 (54.5) | 0.342 |
| Mean score ± SD | 0.68 ± 0.32 | 0.71 ± 0.29 | 0.48 ± 0.40 | 0.091 |
| **TB awareness** |  |  |  |  |
| Q24 Prevalence of TB | 14 (25.9) | 28 (36.4) | 1 (9.1) | 0.124 |
| Q25 Incidence of TB | 37 (68.5) | 56 (72.7) | 9 (81.8) | 0.649 |
| Q26 Specificity of chest x-ray for TB | 13 (24.1) | 20 (26.0) | 2 (18.2) | 0.848 |
| Mean score ± SD | 0.40 ± 0.27 | 0.45 ± 0.31 | 0.36 ± 0.23 | 0.447 |

*Significant at p < 0.05

Abbreviations: CXR, Chest x-ray; CKD, Chronic kidney disease; EGD, Esophagogastroduodenoscopy; TB, Tuberculosis; SD, standard deviation

The knowledge questions (Q) from the questionnaire are listed below. Each correct answer scored 1 point, while 0 points were awarded for incorrect answers. The questions are listed in the Appendix 1.

**Table 3.** The respondents’ scores for the chest x-ray attitude questions

| **Factors affecting physician’s decision regarding preoperative CXRs** | **Mean score ±** **SD** | | | | **p-value** |
| --- | --- | --- | --- | --- | --- |
|  | **Anesthesiologists**  **(n = 54)** | | **Surgeons**  **(n = 77)** | **Gastroenterologists**  **(n = 11)** |  |
| **Patient’s age** | | | | | |
| Q27.6 Over 45 years old | 3.17 ± 1.14 | 3.45 ± 1.33 | | 3.82 ± 1.17 | 0.208 |
| **Low-risk surgery and health concerns** | | | | | |
| Q27.2 Heart disease | 4.72 ± 0.45 | 4.53 ± 0.91 | | 4.73 ± 0.47 | 0.314 |
| Q27.3 Chronic obstructive pulmonary disease | 4.52 ± 0.64 | 4.48 ± 0.98 | | 4.64 ± 0.51 | 0.839 |
| Q27.4 Upper respiratory tract infection | 3.80 ± 1.28 | 3.90 ± 1.28 | | 3.45 ± 1.29 | 0.555 |
| Q27.5 Smoking | 3.96 ± 0.85 | 4.03 ± 1.11 | | 4.18 ± 0.75 | 0.792 |
| Mean score ± SD | 4.25±0.57 | 4.23±0.90 | | 4.25±0.56 | 0.992 |
| **Medicolegal and work-policy issues** | | | | | |
| Q29.1 Avoidance of case cancellation | 3.20 ± 1.07 | 3.64 ± 1.41 | | 3.00 ± 1.27 | 0.090 |
| Q29.3 Prevention of risks or complications | 3.91 ± 0.98 | 3.83 ± 1.08 | | 3.55 ± 1.04 | 0.574 |
| Q29.4 Prevention of prosecution | 3.41 ± 1.06 | 3.29 ± 1.39 | | 3.18 ± 0.87 | 0.793 |
| Q29.5 Following the hospital’s policy | 2.93 ± 0.89 | 2.79 ± 1.40 | | 2.55 ± 0.52 | 0.586 |
| Mean score ± SD | 3.36±0.79 | 3.39±1.03 | | 3.07±0.68 | 0.563 |
| **TB awareness** | | | | | |
| Q27.1 History of TB infection | **4.89 ± 0.32** | **4.58 ± 0.95** | | **4.91 ± 0.30** | **0.048*** |
| Q28.2 For surveillance in hypertensive patient | 3.22 ± 1.21 | 2.95 ± 1.34 | | 3.36 ± 0.92 | 0.356 |
| Q29.2 For surveillance in treated supra ventricular tachycardia patient | 3.09 ± 1.10 | 2.92 ± 1.35 | | 3.09 ± 1.30 | 0.724 |
| Q30.1 Avoidance of case cancellation in patient with history of TB infection | **3.81 ± 1.15** | **4.21 ± 1.10** | | **3.45 ± 1.44** | **0.044*** |
| Q30.2 For surveillance in patient with history of TB infection | 4.15 ± 1.05 | 4.29 ± 1.01 | | 4.64 ± 0.51 | 0.322 |
| Q30.3 Prevention of risks or complications in patient with history of TB infection | 3.96 ± 0.91 | 4.05 ± 1.11 | | 3.82 ± 1.17 | 0.744 |
| Q30.4 Prevention of prosecution in patient with history of TB infection | 3.63 ± 1.05 | 3.43 ± 1.20 | | 3.27 ± 1.9 | 0.492 |
| Q30.5 Following the hospital’s policy in patient with history of TB infection | 3.20 ± 0.94 | 2.84 ± 1.35 | | 2.91 ± 1.04 | 0.231 |
| Mean score ± SD | 3.75 ± 0.70 | 3.66 ± 0.73 | | 3.68 ± 0.60 | 0.788 |

*Significant at p < 0.05

Abbreviations: CXR, Chest x-ray; TB, Tuberculosis; SD, standard deviation

The attitude questions (Q) from the questionnaire are listed below. Each correct answer scored 1 point. The answers were graded and scored according to the level of importance, namely, “extremely important” (5), “very important” (4), “moderately important” (3), “slightly important” (2), and “very slightly important” (1). The questions are listed in the Appendix 1.

**Table 4.** Subgroup analysis of the respondents’ chest x-ray knowledge answers

| **Variables** | **n** | **% with correct answers** | | | | | | | | |
| --- | --- | --- | --- | --- | --- | --- | --- | --- | --- | --- |
|  |  | **Patient’s age** | | | **Low-risk surgery and health concerns** | | | **TB awareness** | | |
|  |  | **Q9** | **Q10** | **Q12** | **Q14** | **Q17** | **Q20** | **Q24** | **Q25** | **Q26** |
| **Sex** |  |  |  |  |  |  |  |  |  |  |
| Female | 75 | 20.0 | 88.0 | 94.7 | 66.7 | 69.3 | 68 | 30.7 | 66.7 | 29.3 |
| Male | 67 | 23.9 | 89.6 | 85.1 | 68.7 | 58.2 | 76.1 | 29.9 | 77.6 | 19.4 |
| p-value |  | 0.685 | 0.797 | 0.088 | 0.858 | 0.220 | 0.351 | 1.000 | 0.191 | 0.180 |
| **Position** |  |  |  |  |  |  |  |  |  |  |
| Resident | 123 | 20.3 | 90.2 | 91.2 | 69.9 | 64.2 | 74.8 | 30.1 | 71.5 | 24.4 |
| Fellow | 19 | 31.6 | 78.9 | 84.2 | 52.6 | 63.2 | 52.6 | 31.6 | 73.7 | 26.3 |
| p-value |  | 0.369 | 0.232 | 0.402 | 0.186 | 1.000 | 0.057 | 1.000 | 1.000 | 1.000 |
| **Department** |  |  |  |  |  |  |  |  |  |  |
| Anesthesiology | 54 | 7.4 | 88.9 | 92.6 | 57.4 | 75.9 | 70.4 | 25.9 | 68.5 | 24.1 |
| Surgery | 77 | 29.9 | 88.3 | 89.6 | 79.2 | 57.1 | 75.3 | 36.4 | 72.7 | 26.0 |
| Gastrointestinal Medicine | 11 | 36.4 | 90.9 | 81.8 | 36.4 | 54.5 | 54.5 | 9.1 | 81.8 | 18.2 |
| p-value |  | **0.004*** | 0.967 | 0.536 | **0.002*** | 0.069 | 0.342 | 0.124 | 0.649 | 0.848 |
| **Preoperative CXR-course attendance** |  |  |  |  |  |  |  |  |  |  |
| Yes | 68 | 27.0 | 85.1 | 90.5 | 66.2 | 63.5 | 70.3 | 27.9 | 70.6 | 23.5 |
| No | 74 | 16.2 | 92.6 | 89.7 | 69.1 | 64.7 | 73.5 | 32.4 | 73.0 | 25.7 |
| p-value |  | 0.155 | 0.190 | 1.000 | 0.724 | 1.000 | 0.711 | 0.588 | 0.825 | 0.846 |
| **Key individual for ordering CXRs** |  |  |  |  |  |  |  |  |  |  |
| Yes | 106 | 13.9 | 88.9 | 100 | 61.1 | 75.0 | 55.6 | 29.2 | 61.1 | 21.7 |
| No | 36 | 24.5 | 88.7 | 86.8 | 69.8 | 60.4 | 77.4 | 33.3 | 75.5 | 33.3 |
| p-value |  | 0.244 | 1.000 | **0.021*** | 0.410 | 0.159 | **0.018*** | 0.677 | 0.133 | 0.183 |

*Significant at p < 0.05

Abbreviations: CXR, Chest x-ray

The knowledge questions (Q) from the questionnaire are listed below. Each correct answer scored 1 point, while 0 points were awarded for incorrect answers. The questions are listed in the Appendix 1.

**Table 5.** Subgroup analysis of the respondents’ chest x-ray attitude answers

| **Variable** | **n** | **% with poor attitude** | | | | | | | | | | | | | | | | |
| --- | --- | --- | --- | --- | --- | --- | --- | --- | --- | --- | --- | --- | --- | --- | --- | --- | --- | --- |
|  |  | **Patient’s age** | **Low-risk surgery and**  **health concerns** | | | | **Medicolegal andwork-policy issues** | | | | **TB awareness** | | | | | | | |
|  |  | **Q27.6** | **Q27.2** | **Q27.3** | **Q27.4** | **Q27.5** | **Q29.1** | **Q29.3** | **Q29.4** | **Q29.5** | **Q27.1** | **Q28.2** | **Q29.2** | **Q30.1** | **Q30.2** | **Q30.3** | **Q 30.4** | **Q 30.5** |
| **Sex** |  |  |  |  |  |  |  |  |  |  |  |  |  |  |  |  |  |  |
| Female | 75 | 40.0 | 94.7 | 89.3 | 62.7 | 65.3 | 45.3 | 70.7 | 44.0 | 28.0 | 94.7 | 41.3 | 38.7 | 64.0 | 81.3 | 62.7 | 46.7 | 32.0 |
| Male | 67 | 61.2 | 95.5 | 91.0 | 64.2 | 77.6 | 59.7 | 65.7 | 52.2 | 25.4 | 94.0 | 40.3 | 38.8 | 82.1 | 83.6 | 82.1 | 50.7 | 31.3 |
| p-value |  | **0.018** | 1.000 | 0.785 | 0.863 | 0.138 | 0.095 | 0.589 | 0.400 | 0.850 | 1.000 | 1.000 | 1.000 | **0.023** | 0.827 | **0.015** | 0.737 | 1.000 |
| **Position** |  |  |  |  |  |  |  |  |  |  |  |  |  |  |  |  |  |  |
| Resident | 123 | 48.8 | 95.1 | 89.4 | 64.2 | 70.7 | 54.5 | 70.7 | 50.4 | 29.3 | 94.3 | 40.7 | 39.8 | 74.0 | 81.3 | 71.5 | 50.4 | 34.1 |
| Fellow | 19 | 57.9 | 94.7 | 94.7 | 57.9 | 73.7 | 36.8 | 52.6 | 31.6 | 10.5 | 94.7 | 42.1 | 31.6 | 63.2 | 89.5 | 73.7 | 36.8 | 15.8 |
| p-value |  | 0.623 | 1.000 | 0.693 | 0.616 | 1.000 | 0.217 | 0.122 | 0.145 | 0.101 | 1.000 | 1.000 | 0.616 | 0.407 | 0.527 | 1.000 | 0.329 | 0.183 |
| **Department** |  |  |  |  |  |  |  |  |  |  |  |  |  |  |  |  |  |  |
| Anesthesiology | 54 | 44.4 | 100 | 92.6 | 64.8 | 70.4 | 44.4 | 74.1 | 51.9 | 27.8 | 100 | 42.6 | 38.9 | 64.8 | 75.9 | 68.5 | 53.7 | 38.9 |
| Surgery | 77 | 50.6 | 90.9 | 87.0 | 63.6 | 70.1 | 61.0 | 66.2 | 48.1 | 29.9 | 89.6 | 39.0 | 39.0 | 81.8 | 84.4 | 74.0 | 46.8 | 28.6 |
| Gastrointestinal Medicine | 11 | 72.7 | 100 | 100 | 54.5 | 81.8 | 27.3 | 54.5 | 27.3 | 0.0 | 100 | 45.5 | 36.4 | 45.5 | 100 | 72.7 | 36.4 | 18.2 |
| p-value |  | 0.229 | **0.045*** | 0.414 | 0.803 | 0.813 | **0.037*** | 0.377 | 0.340 | 0.096 | **0.035*** | 0.836 | 1.000 | **0.009*** | 0.137 | 0.811 | 0.536 | 0.333 |
| **Preoperative CXR course attendance** |  |  |  |  |  |  |  |  |  |  |  |  |  |  |  |  |  |  |
| Yes | 68 | 57.4 | 95.9 | 90.5 | 62.2 | 68.9 | 50.0 | 67.6 | 47.3 | 23.0 | 94.6 | 40.5 | 36.5 | 68.9 | 78.4 | 71.6 | 47.3 | 31.1 |
| No | 74 | 43.2 | 94.1 | 89.7 | 64.7 | 73.5 | 54.4 | 69.1 | 48.5 | 30.9 | 94.1 | 41.2 | 41.2 | 76.5 | 86.8 | 72.1 | 50.0 | 32.4 |
| p-value |  | 0.130 | 0.710 | 1.000 | 0.862 | 0.582 | 0.618 | 0.859 | 1.000 | 0.334 | 1.000 | 1.000 | 0.608 | 0.351 | 0.270 | 1.000 | 0.867 | 1.000 |
| **Key individual for ordering CXRs** |  |  |  |  |  |  |  |  |  |  |  |  |  |  |  |  |  |  |
| Yes | 106 | 55.7 | 97.2 | 91.7 | 61.1 | 63.9 | 41.7 | 61.1 | 41.7 | 25.0 | 97.2 | 44.4 | 41.7 | 63.9 | 80.6 | 61.1 | 55.6 | 36.1 |
| No | 36 | 33.3 | 94.3 | 89.6 | 64.2 | 73.6 | 55.7 | 70.8 | 50.0 | 27.4 | 93.4 | 39.6 | 37.7 | 75.5 | 83.0 | 75.5 | 46.2 | 30.2 |
| p-value |  | **0.033** | 0.679 | 1.000 | 0.842 | 0.291 | 0.178 | 0.305 | 0.443 | 0.831 | 0.680 | 0.696 | 0.696 | 0.199 | 0.801 | 0.133 | 0.343 | 0.538 |

*Significant at p < 0.05

Abbreviations: CXR, Chest x-ray

The attitude questions (Q) from the questionnaire are listed below. Each correct answer scored 1 point. The answers were graded and scored according to the level of importance, namely, “extremely important” (5), “very important” (4), “moderately important” (3), “slightly important” (2), and “very slightly important” (1). The questions are listed in the Appendix 1.

**Table 6.** Correlations between the total scores for the knowledge and attitude questions

| **Correlations** | | **Spearman’s**  **correlation coefficient** |
| --- | --- | --- |
| **Knowledge** | **Attitude** |  |
| Patient’s age | Patient’s age | -0.201* |
| Low-risk surgery and health concerns | Low-risk surgery and health concerns | 0.311** |
| TB awareness | TB awareness | 0.046 |
| Low-risk surgery and health concerns | Medicolegal and work-policy issues | 0.257* |
| **Attitude** | **Attitude** |  |
| Low-risk surgery and health concerns | Medicolegal and work-policy issues | 0.335** |

**Significant at p < 0.001

*Significant at p < 0.05

Abbreviations: CXR, Chest x-ray; TB, Tuberculosis

**Table 7.** Percentage of respondents with good and poor knowledge and attitudes for each set of correlations

| **Correlations** | | **n (%)** | | | | | |
| --- | --- | --- | --- | --- | --- | --- | --- |
| **Knowledge** | **Attitude** | **Good K** | **Good A** | **Good K**  **Good A** | **Good K**  **Poor A** | **Poor K**  **Good A** | **Poor K**  **Poor A** |
| Patient’s age | Patient’s age | 121 (85.21) | 71 (50.00) | 58 (40.85) | 63 (44.37) | 13 (9.15) | 8 (5.63) |
| Low-risk surgery and health concerns | Low-risk surgery and health concerns | 106 (74.64) | 10 (7.04) | 6 (4.22) | 100 (70.42) | 4 (2.82) | 32 (22.54) |
| TB awareness | TB awareness | 49 (34.51) | 43 (30.28) | 17 (11.97) | 32 (22.54) | 26 (18.31) | 67 (47.18) |
| Low-risk surgery and health concerns | Medicolegal and work-policy issues | 106 (74.64) | 61 (42.95) | 41 (28.87) | 65 (45.77) | 20 (14.08) | 16 (11.27) |
| **Attitude 1** | **Attitude 2** | **Good A1** | **Good A2** | **Good A1**  **Good A2** | **Good A1**  **Poor A2** | **Poor A1**  **Good A2** | **Poor A1**  **Poor A2** |
| Low-risk surgery and health concerns | Medicolegal and work-policy issues | 10 (7.04) | 61 (42.95) | 6 (4.22) | 4 (2.82) | 56 (39.44) | 76 (53.52) |

Abbreviations: Good K, Good knowledge; Good A, Good attitudes; Poor K, Poor knowledge; Poor A, Poor attitudes; TB, Tuberculosis
